# Inpatients experiences about the impact of traumatic stress on eating behaviors: an exploratory focus group study

**DOI:** 10.1186/s40337-021-00480-y

**Published:** 2021-09-26

**Authors:** Grethe Emilie Roer, Heidi Hurlen Solbakken, Dawit Shawel Abebe, Jan Olav Aaseth, Ingeborg Bolstad, Lars Lien

**Affiliations:** 1 Norwegian National Advisory Unit on Concurrent Substance Abuse and Mental Health Disorders, Innlandet Hospital Trust, P.O. Box 104, 2381 Brumunddal, Norway; 2 Department of Nursing and Health Promotion, Oslo Metropolitan University, St. Olavs plass, P.O. Box 4, 0130 Oslo, Norway; 3 Research Department, Innlandet Hospital Trust, P.O. Box 104, 2381 Brumunddal, Norway; 4 Faculty of Social and Health Sciences, Inland Norway University of Applied Sciences, P.O. Box 400, 2418 Elverum, Norway; 5 Friskstiftelsen, Hamarvegen 112, 2406 Elverum, Norway

**Keywords:** Stress disorders, Post-traumatic, Feeding behavior, Appetite, Psychological, Feeding and eating disorders, Focus groups, Inpatients

## Abstract

**Background:**

Unhealthy changes in eating behavior among people experiencing trauma have been observed. There is, however, a lack of in-depth knowledge regarding the impact of the after effects of traumatic life experiences on eating behavior. Because eating behavior represents important components for promotion and maintenance of good health throughout life, this study aimed to explore inpatients’ lived experiences of the impact of traumatic stress on eating behavior.

**Method:**

Thirteen female and two male inpatients (age range 28–62 years), recruited from a psychiatric clinic in Norway, participated in this qualitative explorative focus group study. The data analysis was performed using systematic text condensation.

**Results:**

The results in the present study describe the participants’ experiences about the impact of traumatic stress on their eating behavior. Their discussions and descriptions disclosed problems that could be summarized into four main themes: “experiencing eating behaviors as coping strategies”; “experiencing being addicted to food and sweets”; “experiencing eating behaviors controlled by stress and emotions”; and “experiencing lack of appetite and reduced capacity to plan and prepare meals”.

**Conclusion:**

Traumatic stress can impact eating behavior in several complex ways that over time may cause adverse health consequences. The results add to an important understanding of changes in eating behavior that might appear in people struggling to cope with the after effects of traumatic life experiences to the existing literature. To better understand the complexity of how traumatic experiences may impact eating behavior, this knowledge is important and useful for health professionals offering support to those who experience struggling with eating behavior after traumatic experiences.

## Plain English Summary

This study aimed to explore trauma-exposed inpatients experiences about the impact of traumatic stress on eating behavior. Thirteen female and two male inpatients with a history of trauma, recruited from a psychiatric clinic in Norway, participated in this qualitative explorative focus group study. The results in the present study describe the participants’ experiences about the impact of after effects of traumatic experiences on eating behaviors. The findings are summarized into four main themes: “experiencing eating behaviors as coping strategies”; “experiencing being addicted to food and sweets”; “experiencing eating behaviors controlled by stress and emotions”; and “experiencing lack of appetite and reduced capacity to plan and prepare meals”. The results contribute to the existing literature and provide an important understanding of changes in eating behavior that might appear in people struggling with traumatic stress after traumatic experiences. This knowledge is important and useful for health professionals offering help to those struggling with their eating behavior after traumatic experiences.

## Introduction

Traumatic stress, which refers to physical and psychological reactions occurring in varying degrees after traumatic experiences, characterized by symptoms such flashbacks; nightmares; avoidance; a sense of “numbness” and emotional blunting; anhedonia and acute burst of fear or panic [1], can cause changes in eating behavior [2, 3]. Studies addressing eating behavior among people after traumatic experiences, suggest that symptoms related to posttraumatic stress disorder (PTSD) are associated with consumption of soda and fast food, and unhealthy dieting behavior [4]. Eating behavior, defined by LaCaille [5] as “a broad term that encompasses food choice and motives, feeding practice, dieting, and eating-related problems such as obesity, eating disorders (ED), and feeding disorders”, represents important components for promotion and maintenance of good health throughout life [6].

Traumatic experiences might be a risk factor for developing disordered eating behavior. Childhood trauma are found to be a risk factor for developing EDs [7–9], and a history of trauma is associated with greater symptoms of EDs [10]. Unlike trauma patients without PTSD, those with PTSD report a greater number of symptoms of EDs [10]. In a study of male and female veterans, the severity of PTSD and depression were associated with greater symptoms of bulimia nervosa (BN) and binge-eating disorder (BED) [11].

Emotional regulation difficulties and emotional eating is suggested as mediators in the association between binge-eating and post-traumatic stress symptoms [12], and emotional dysregulation and dissociation have been suggested as mediators between childhood-trauma and eating psychopathology [13–15]. In a qualitative study by Breland et al. [16], the participants reported disordered eating, such as binge-eating, as a means of short-term relief from trauma-related negative affect. Emotionally conditioned eating is also considered linked to regulation of stress [17], while stress, in turn, has been found to reduce emotional and behavioral control and increase impulsivity [18], which thus can lead to overeating. When feeling stressed, the brain expresses both a strong desire to eat and a reduced ability to inhibit eating [19]. Food and sweets are easy to access, and could make us feel more relaxed or comforted [20], in a better mood and less stressed [21–23]. However, whereas chronic stress increases the desire for sweet and tasty high-fat foods [24], acute stress can, in turn, reduce appetite [25].

In recent years the differentiation between, and potential overlap with, food addiction and EDs such as BN and BED have been discussed in the literature [26]. Result from a cross-sectional study, consisting of 49,408 female nurses, found that higher levels of PTSD-symptoms were associated with increased levels of food addiction [27], and a separate study found that childhood trauma was associated with both food addiction and binge eating [28]. In addition, results from the systematic review of Pursey et al. [29] showed higher prevalence of food addiction in people with disordered eating. The concept of food addiction is, however, disputed [30].

Most previous research addressing changes in eating behaviors after traumatic experiences have been quantitative. There is still a lack of in-depth knowledge about the impact of lived experience with traumatic stress on eating behavior. Therefore, the aim of this qualitative study was to explore the experiences of inpatients with severe trauma-related mental health disorders and the impact this had on their eating behaviors.

## Methodology

### Design

The present study is the first part of a research project exploring the occurrence of, and possible risk factors for, metabolic syndrome and type 2 diabetes mellitus after traumatic experiences. To explore and describe the subjective experiences of how traumatic stress impacts on eating behavior, we applied a explorative qualitative focus group design with an inductive approach [31]. Using focus group interviews are an effective and suitable method for exploring specific problem areas. Despite this topic being potentially sensitive, a focus group can still be a safe arena in which to share and discuss experiences about such topics [32]. In addition, the discussions and interactions between participants, depending on the success of the particular groups, will be less influenced by the researcher, and may generate new questions and highlight important aspects the researcher was unable to anticipate [32].

### Recruitment and participants

Participants were recruited from a psychiatric clinic in Norway which offers inpatient treatment to adults (> 18 years) with severe mental disorders such as PTSD, complicated with anxiety and depression. The clinic has a total of 25 beds where the patients live and receive intensive psychological treatment for a period of three months. The first contact with the informants was made by nurses and psychologists at the clinic who provided information about the study and obtained written consent. Thereafter, the first author contacted the recruited participants, gave information about time and place for the focus group interviews, number of participants in the focus group, and repeated the purpose of the study.

Thirteen female and two male participants (age ranged 28–62 years) from the south-eastern part of Norway who received treatment at the clinic participated in the study. The mean age of the participants was 44 years (SD = 9.6). All participants in the study had a history of traumatic experiences and had received treatment for severe trauma-related disorders in the specialist health service for many years. According to the criteria for receiving treatment at the clinic, the participants had no current severe symptoms of psychosis, severe ED, acute suicidality, substance abuse, self-harm or violent behaviors.

### Data collection

We conducted three focus group interviews with five participants in each group. Originally, we aimed for 6–8 participants in each, but due to restrictions at the clinic related to the Covid-19 pandemic, only five participants were recruited for each focus group interview. Number of focus group interviews was chosen according to the model of *information power* [33]. After completion of the third focus group interview, no new findings emerged, only confirmations of findings from the first two interviews.

The focus group interviews were conducted during daytime and within familiar premises at the clinic. Each interview lasted for 90 min and were conducted only once with each group. The second author led the interviews by acting as the moderator. The first author had a more reclusive role, primarily serving as an observer and administrator. Coffee, tea, water and juice were served during the interviews. Two tape recorders were used to record the interviews. In addition, the observer took notes to capture body language, mood, reactions and interaction between participants.

An interview guide was developed for the interviews, consisting of open-ended questions which would serve as a support for the moderator. The moderator invited the participants to discuss the following topics:

- What thoughts spring to mind when you hear me say stress or stress in everyday life?
- What thoughts spring to mind when you hear me say nutrition or food in general?
- Does stress impact upon your eating behavior? If so, how?
- Have previous traumas had an impact on, or led to changes in, eating behavior? If so, how?
- Changes in eating behavior, did they contribute to managing perceived stress in everyday life or any discomfort connected to your experience(s)?

In this study, “trauma” is referred to as a subjective experience, and not specific traumatic events [34]. Type of traumatic events were not systematically examined, but some of the participants reported traumatic experiences in childhood, adolescence and adulthood. Whether the participants had some form of EDs or not was not mapped before the focus group interviews, but some of the participants reported a history of previous ED, such as anorexia nervosa, BN, or BED. For the sake of the informants’ privacy, no further sociodemographic information was collected. The participants were also informed that there was no intension to delve into personal traumatic experience(s).

### Ethical aspects

The study was assessed and approved by the Regional Committees for Medical and Health Research Ethics (REK) (#78587 in REK South-East B), the Norwegian Centre for Research Data (NSD) (#616642) and the Data Protection Officer at Innlandet Hospital Trust (#135535).

The participants were informed that participation in the study was completely voluntary, and that at any time they could withdraw their consent without reason. Moreover, the participants were informed of their free access to read the transcribed material about themselves. The participants shared sensitive topics and stories, therefore caretaking of each informant, including anonymization, has been given a high priority.

Participation in focus group interviews could cause strain on the individual through hearing stories and experiences of other participants. We, therefore, prepared a contingency plan prior to the focus group interviews, intended to prevent and take care of potentially vulnerable and stressful situations.

### Data analysis

*Systematic text condensation* (STC) [31] was used to analyze the data. This approach is a descriptive and exploratory method, suitable for thematic interdisciplinary analysis of various types of qualitative data, such as data from interviews. The method represents a pragmatic approach, consisting of the following four steps: (1) *total impression—from chaos to themes;* (2) *identify and sort meaning units—from themes to codes*; (3) *condensation—from code to meaning*; (4) *synthesizing—from condensation to descriptions and concepts* [31].

Directly, after all three of the focus group interviews, the first and second author discussed and reflected upon the main impressions of the interviews. The first author transcribed each interview, word by word including body language and other linguistic information such as pauses, sighs, crying, and listened through several times to familiarize herself with the material. Between the second and third interview, the first and second author read, separately, the transcribed material. Thereafter, the first, second and last author met to discuss the overall impression and tentative themes, whereupon it was agreed to conduct a third interview. After completing all three interviews, the research team, including peer researchers with service-use experience, met and discussed the overall impression and tentative themes from the interviews. Then, the first and second author, separately, selected out meaningful units and discussed which to keep for further coding and condensation. The first author wrote ‘artificial quotations’ and from these selected ‘authentic illustrative quotations’ [31]. The second author and an independent translator, together with the first author, translated the authentic illustrative quotations. Finally, the results were discussed with the research team and peer researchers. Discussing the data material and results with the peer researchers was meaningful and enriching, and brought new perspectives and insight into the data material, providing a broader and deeper understanding [35].

According to the analytical method of Malterud [31], the focus was to present the subjective experiences of the patients, rather than possible underlying meanings. The analytical process has been inductive, dynamic and flexible, by going back and forth when evaluating the research topic, interview guide, codes and themes [31]. Table 1 illustrates the steps in the analysis process.

**Table 1:** Illustration of the steps in systematic text condensation

Step 1:	Step 2:	Step 3:	Step 4:
Preliminary theme	Meaning units (direct quotation from a participant)	Condensed description	Final theme and analytical text
Emotional regulation	"I tend to be more drawn to food items that I think will give me a sense of comfort, or treats, or like, "I'm having a bad day, so I deserve this and that". Perhaps in some periods, every day is a bad day. So I eat more, like loads of pizza for example."	Drawn to food that comfort. Eat loads of pizza in periods when every day is a bad day	Theme: Experiencing eating behaviors as coping strategies
			Analytical text: […] seek out food that comforted them […]
Lack of energy	"Ehm, for me, it mainly has to do with time and energy. When I am stressed, I do not have neither the time nor the energy to eat what is good for me."	Cooking and eating healthy both have to do with surplus time and energy. Have neither the time nor the energy when stressed out	Theme: Experiencing lack of appetite and reduced capacity to plan and prepare meals
			Analytical text: […] not having time or energy to plan and prepare meals […]

## Results

Based on the data material from the focus group interviews, four main themes were identified which described the participants’ experiences of how traumatic stress impacted on their eating behavior: (1) experiencing eating behaviors as coping strategies; (2) experiencing being addicted to food and sweets; (3) experiencing eating behaviors controlled by stress and emotions, and; (4) experiencing lack of appetite and reduced capacity to plan and prepare meals. The content of each theme is described in the paragraphs below.

### Experiencing eating behaviors as coping strategies

The participants discussed and described eating behavior, e.g. binge eating, increased intake of fast food, sweets and salty snacks, restrictive and controlled eating motivated by fear and a strong urge to regulate or avoid discomfort related to stress and emotions, especially in relation to the previous trauma. As one female participant stated: “It is so easy to just eat”. The participants described a fear of emotions and traumatic memories that might arise if they did not eat. One participant explained:

Emotions for me, they express themselves in a physical way. I feel it in my stomach. […] I am scared of what will come, so I use food to suppress it. It is so uncomfortable. I do not know what it represents or… Or I am starting to realize, but what it represents is in the past and it is so disturbing for me. I cannot deal with it at the moment, so I just have to eat. […] The quantity and what I actually eat has varied throughout life, but I eat, and mainly sweets.

For a few participants, certain types of food, or the eating situation itself, triggered traumatic memories, gave feelings of bad conscience or generated full anxiety. The previous trauma made it difficult to sustain a varied diet and led to increased consumptions of fast food, salty snacks and sweets. Their eating behavior became, in a way, a form of strategy to avoid anxiety, panic or bad conscience. As one participant explained, “[…] then [after the trauma] I start throwing up and struggle a lot with food. In periods I binge-eat, which gives me bad conscience and I stop eating”. Some also hid their consumption of sweets and salty snacks, because of the fear they carried from the experienced trauma. One participant explained:

Some foods I can eat. Other foods just trigger my anxiety and restlessness and fear and chaos. [Hesitates] […] And that makes it very difficult today, if someone serves you some of this food […]. I will have to drown the food in something I like, like sour cream or gravy, to camouflage the taste, camouflage the memories. Instead one ends up eating loads of “bad” food, because this is food you manage to eat. That, in turn, resulting in you gaining weight.

Another theme discussed and described by the participants was the function of food, sweets or salty snacks for self-comforting. They would seek out food that comforted them or calmed emotions; eating a bag of potato chips instead of asking for a hug, or in tough times, eating pizza, salty snacks or sweets because they felt they deserved it. As one said, “I feel drawn to food items that I think will give me a sense of comfort”.

Other participants described restrictive and controlled eating, *or* purge behavior e.g., by only eating unprocessed food; avoid eating potatoes, rice and pasta, or unhealthy overeating. As one explained, “I’m scared of what will happen emotionally if I eat more than my normal amount”. Other participants explained that eating too much or unhealthy foods affected their body-image negatively, or made them feel hypo-arousal which then triggered traumatic memories. In contrast, another participant experienced her body-image triggered consumption of unhealthy food because she forced herself into eating unhealthy to become as ugly as she felt, while another participant could use food as a form of self-punishment, explaining the following:

[…] Since I was quite young, in addition to other types of self-harm, I have used… I could eat chocolate really fast and then purge it immediately after. I remember it as a way of keeping control. I also felt more alert and present afterwards. So food goes in, but it has to come out as well. This I could control (laughter), I couldn't control the other things.

Both binge eating and not eating at all was explained, by two participants, as a form of self-medication that made the “chaos” inside their head a bit calmer. “You can take a pill or you can stop eating, it will be a bit of the same”, one explained. Another participant described:

The urge to suppress emotions trumps the urge to eat normal food. I call it my medication- binging on the wrong kind of food. But of course, it is not a viable solution.

In the absence of other coping strategies, binge-eating or restrictive eating developed in childhood or adolescence as a coping strategy to “survive” painful emotions or forget severe traumatic experiences, was described by some participants. “If an adult had asked me as a child why I started eating a lot of food, perhaps things could have been discovered, and other things prevented”, one explained. Another participant explained:

It has just been a way to survive […]. It worked as a survival mechanism when I was young, but then it has just persisted because the emotions are sort of stuck in time. I still cannot relate to what happened. Do not want to know. So, I just have to go on, with the coping mechanisms. It keeps the emotions and discomfort at a distance. […]. It turns out it has had a much larger and deeper emotional function than I have realized. [Sighs] […]

### Experiencing being addicted to food and sweets

In all three focus groups, some participants discussed and compared food, sweets and salty snacks with addiction and abuse of alcohol and drugs, and the complexity of why people might get addicted. For example, one participant explained:

Food, in a way, is harmless, because it is acceptable as opposed to intoxication, alcohol or drugs…. Even though it has the same function, it is sort of okay because it’s legal and isn’t considered to be a serious problem. Being underweight or obese are seen as problems, but the problem is more complex and multifaceted, and is something I have trivialized my whole life (sighs). But in fact, there is so much more to it.

These participants experienced that it was especially difficult being addicted to food, compared to alcohol, because they still had to eat and relate to it several times a day. As one explained, “I might be able to hold my pantry clean for junk food, and avoid buying it if I’m steadfast enough, but I still have to deal with it in my everyday life you know”. In the context of addiction, a few participants also discussed how they experience being met by others if saying “no thanks” to sweets. As one explained, “Contrary to the positive feedback I would have got if I said no thanks to wine as a former alcoholics, I am met with questions and reluctance if I say no thanks to cake since because I am a former sugarholic”. About the experience of sugar addiction, another explained, “I don’t eat chocolate because I get so addicted. […], and I think I’m predisposed to be addicted”.

In addition, the participants discussed how stressful periods with general hardship, or months of trauma therapy, made them crave especially for sugar, salt and fat. Several kilos of sweets could be consumed, and some described compensatory eating behaviors, such as purge behavior or restrictive eating to compensate for the intake of sugar. As one explained, “And then I restrict, only eating a minimal amount, really forcing my body back to my starting weight so I can go back to eating sweets again”. Some participants could lose their appetite, but still crave for sweets and salty snacks. As one explained, “It’s a large craving for the sweet and salty snacks, like Smash and such tasty snacks. It’s impossible to eat just one”. Another said that she could even dream about sweet buns, and just had to buy buns, because not buying them felt wrong.

Some of the participants also shared that they were hiding drinking soda or their intake of sweets and salty snacks. Nobody ever saw them eating or drinking it, and that eating so much sweets led to isolation because of bad conscience, shame and guilt. One participant explained:

The bad conscience that comes after binging makes you isolate yourself and not leave the house. It’s a consequence from binging, a ripple effect.

Despite feeling their body becoming unwell consuming amounts of fast food, and knowing about the unhealthy consequences, some participants reported continuing to consume as they felt the consequences were insignificant in comparison to the strong need to suppress emotions and stress. As one stated, “It just has to be done”.

### Experiencing eating behaviors controlled by stress and emotions

In all three focus groups, participants discussed the experience that stress and emotions led to impulsive and uncontrolled food choices which resulted in binge-eating or increased intake of fast food, sweets and salty snacks. “It’s not something I feel that I control. I’m controlled by stress and emotions”, one explained. Another participant shared feelings of desperation in stressful times and difficulties in thinking clearly:

[…] When I’m stressed and desperate I make really bad choices (crying) […] I can’t think clearly, but rationally I know it's not a good choice, but I end up making it anyway. Later on, when I have the time and the state of mind to reflect upon it, I don't understand why I'm choosing the way I do.

For many participants, it became clearer for them, in the focus group discussion, how stress actually affected their eating behavior unconsciously. As one expressed, “[…] it just became clear now, because I’m talking about it, but otherwise just happens by itself”. Another told that she experienced eating sweets without knowing it, or that she could eat sweets, and then, suddenly become aware of it, thinking that it was not healthy. She explained the following:

It comes to a point when I’ve been sitting eating sweets, and suddenly it dawns upon me. You just feel bloated and stuff. There are more left, but I’ve come to my senses and realize “this is no good”. [Laughter]. So, I throw it in the trash. I have come to realize I have thrown away loads of money.

For a few participants, even if they made a list before grocery shopping, or aimed not to buy sweets, they got so stressed by all the people, that they ended up buying chocolate anyway. “I can feel my body just moving towards sweet buns and soda, like the body goes on autopilot”, one explained, while another described that she felt the whole world was raging when entering the checkout and seeing all the sweets. Afterwards, they could feel even more stressed because of bad conscience, and some expressed that they did not understand why they made such food choices in stressful situations. To compensate for consuming fast food and sweets, a few participants would punish themselves afterwards with fasting or purging. However, in what they called ‘*better periods’* in their lives, the participants explained that they made more conscious, thoughtful and healthy food choices.

### Experiencing lack of appetite and reduced capacity to plan and prepare meals

Participants discussed their experiences of losing their appetite and feeling of hunger in difficult and stressful periods, and not having time or energy to plan and prepare meals because of stress, anxiety or depression. “I have no appetite these days. It’s like nothing tastes good at all, except from sweets or Cheese Doodles”, one explained, and another that she felt her body became allergic to everything except water or coffee when feeling stressed or anxious. Some described irregular diets and unstructured eating habits. As one explained, “At home, I don't have any routines, at least not regarding meals, you know. I get up late, and have breakfast at 6 pm, right”.

The participants explained that they felt too exhausted thinking about what they should eat, and that it was easier to just resort to easy food solutions, such as junk food, hot-dog, noodles, kebab, pizza, sweet buns, cappuccino, Cheese Doodles, potato chips or sweets. Preparing food at all, could for some also be difficult. As one participant expressed, “When I am extremely exhausted and frail, and life feels terrible, just turning on the oven to heat up a pizza is unbearable”. So, the food had to be easy to prepare, and not time consuming:

It's all about energy. When I'm feeling stressed and don't have the energy or time to cook, I have a lot of cappuccino with hot milk and chocolate buns or take-away meals. Then it's what's simple, and nearby, like Burger King or other take-away shops. There are no healthy take-away options where I live, so it is about accessibility.

On the other side, in days of wellbeing and feeling calm, they felt more appetite, had the time and capacity to both plan and prepare healthy meals, and felt it was easier to actually eat food:

When you are in a better place, it’s physically possible to have a regular meal and keep it inside your body without it hurting or something.

## Discussion

The main finding from the present study is that the participants eating behaviors were severely affected in various complex ways as a result of the experience of stress, resulting in four main themes: experiencing eating behaviors as coping strategies”; “experiencing being addicted to food and sweets”; “experiencing eating behaviors controlled by stress and emotions”; and “experiencing lack of appetite and reduced capacity to plan and prepare meals”. In addition to the participants’ experiences of how traumatic stress impacted eating behaviors, they explained their experiences of *why* traumatic stress impacted their eating behaviors.

### Experiencing eating behaviors as coping strategies

Our results show several ways of how traumatic stress in acute stressful situations, or connected to negative emotions, traumatic memories and restlessness can impact eating behavior. Using words like *survive* illustrates the severity of the suffering and the forces that underlie behavior, which also appears in the participants' use of expressions like “a strong urge” and “scared of what will come”. Results from recent findings suggest that unhealthy eating behavior and diet quality in people who have experienced trauma can be understood by emotional eating, emotional regulation difficulties and emotional dysregulation [12–15, 36, 37]. These findings are similar to the participants’ experiences in this present study, e.g. eating behavior motivated by a need to regulate and suppress stress and emotions. However, our results suggest that in addition to poor regulation, stress and emotions can impact eating behavior in a more restrictive and controlled regime. Controlled eating can be operationalized as the individuals' subjective experience of lack of control over their own lives where controlling their appetite demonstrates that they have regained control [38]. Similar to the results in this study that suggest restrictive and controlled eating are used to avoid or suppress negative emotions, earlier studies have found restrictive eating to be associated with difficulties in emotional regulation [39], and preoccupation with food or anorexic symptoms in order cope with emotions [40, 41]. Further, increased or decreased caloric intake during stress [20], might be explained by the nature of the stressor [42–44].

Previous studies have focused on disordered eating and emotional regulation in people who have experienced trauma [14, 36, 37]. Contributing to the existing literature, this study, reveals that some participants explained their eating behavior as a way to avoid traumatic memories or being reminded of a past trauma by binge-eating, avoiding specific types of food or eating beyond satiety. As far as we know, these findings have not been described in the literature previously. Some of this can may be understood in terms of avoidance of activities reminiscent of the trauma [1], but the findings provide a more in-depth understanding of how and why changes in eating behavior can occur in people who have experienced trauma.

Further, our results suggest that for some participants, eating behavior such as binge-eating or restrictive eating is experienced as a form of self-medication. Food is easy to access, and considering the function it could serve in regulation of mood and stress [20–23], is it not incomprehensible that people suffering after traumatic experiences resort to food. For several of the participants’, using food as a cooping strategy was developed in early childhood, pointing to the importance of awareness of potentially maladaptive eating behaviors that may arise from the inability to manage and regulate stress and negative emotions.

In order for healthcare professionals to understand patients’ eating behaviors as a strategy for regulating and controlling emotions and traumatic stress, it is important to be person-centered and to understand what *motivates* the behavior, i.e. the driving force behind individuals’ behavior. In the light of Hedonistic Motivational Theory [45], the behavior may be motivated by a desire for pleasure and avoiding pain. Several aspects of the participants’ descriptions, e.g., binge-eating, restrictive or controlled eating, seemed to be motivated by a desire to avoiding pain related to, for example, negative emotions and memories from the previous trauma. Similar to previous findings [16], however, several participants expressed that they had not been conscious about how or especially why, traumatic stress actually impacted their eating behavior before the interview, which may indicate that that they are not conscious of their eating behavior or how traumatic stress may impact their eating behavior.

Consequences of maladaptive eating behavior, both consuming high amounts of sweets, salty snack and ultra-processed food, or restrictive eating, might have serious consequences for the individuals’ health. Long-term effects include a number of severe health consequences [46–48], including anorexia nervosa, BN and BED [49, 50].

### Experiencing being addicted to food and sweets

In the participants’ discussion about food, alcohol and drugs, they compared food, sugar, and their eating behavior to drugs and alcohol addiction. The concept of food addiction has been critically discussed in the literature [30], but the engagement of this topic among the participants, might signal the importance of a new approach and understanding of binge-eating and bulimic behavior [26, 30]. Additionally, the participants’ expressions and descriptions of the strong urge and cravings toward ultra-processed food and snacks also illuminate an urge that is stronger than the willpower to avoid such ultra-processed food and snacks. According to the International Statistical Classification of Diseases (ICD-10) (F10–F19) [51], the participants’ descriptions had many similarities with the definition of dependence syndrome, in addition to similarities with food and sugar addiction [52], which can be characterized by difficulties in controlling normal behavior despite negative consequences [53]. For instance, continuing to consume ultra-processed food and snacks despite the consequences for the health; a strong urge or compulsion to eat, and problems controlling intake (use) and quantities. Consuming high levels of sugar, salty snacks and other types of comfort food might be less harmful and more socially accepted than drugs and alcohol. However, it can lead to serious consequences, including obesity and type 2 diabetes mellitus [54–56].

Chronic stress has been found to indicate increased food addiction scores in mice being fed palatable food [57]. If this is the case, and traumatic stress is understood as chronic stress, this might suggest that experiencing subsequent prolonged traumatic stress after trauma can be a risk factor for food and sugar addiction. PTSD in older veterans have been found to be associated with food addiction [58], and a cross sectional study of 49,408 female nurses found increased prevalence of food addiction with the number of lifetime PTSD symptoms [27]. The participants also described compensatory behavior performed to avoid weight gain or feeling of guilt, such as restrictive eating and dieting to lose weight, or vomiting. Compensatory eating behaviors, such as fasting [59], which have a connection to body dissatisfaction and purging [60] have, however, been associated with both disturbed eating and EDs.

In line with earlier findings showing that depression and stress-like mood increases cravings for chocolate [61], our results show that the participants experienced cravings for ultra-processed food and snacks, especially in stressful and hard times. Important to mention that, particularly in the case of high sugar intake, the participants experienced social isolation afterwards because of bad conscience, shame and guilt. Social isolation in turn, can have additional negative impacts on health [62]. Studies in Norway have reported that childhood physical maltreatment is associated with higher risk of perceived social isolation in adulthood [63], and that perceived loneliness is associated with increased consumptions of sugary beverages [64]. This underlines the importance of preventing social isolation and loneliness among people who have experienced trauma, to avoid possible negative consequences on psychological as well as physical health.

### Experiencing eating behaviors controlled by stress and emotions

It appears from our results that some participants experienced their eating behavior to be controlled by stress and emotions or experienced themselves as losing control and awareness over food choices, eating when feeling stressed and overwhelmed by emotions. There is no doubt that the participants experienced being controlled by stress and emotions, which clearly emerges from statements like “it’s not something that I feel that I control”. Apparently, they struggled with high levels of stress in some situations, using words like “desperate”. Previous research supports these observations [18, 19, 65]. When feeling stressed, the brain expresses both a strong desire to eat and a reduced ability to inhibit eating [19].

Traumatic stress can evoke two emotional extremes, either feeling too much (overwhelmed) or too little (numb) emotions [66]. Experiencing being controlled by stress and emotions, can be understood as the participants’ experience being overwhelmed by emotions, which in turn gives an experience of losing control. Further, stress has been found to reduce emotional and behavioral control and increase impulsivity [18], which may lead to overeating. Given that people exposed to trauma, experience loss of control over food because they feel controlled by stress and emotions, treatment may help getting the optimal level of emotions and the regulation of them [66].

Additionally, as in the second theme, participants talked about compensatory eating behaviors [59] since they felt stressed or guilty after eating or binging ultra-processed food, sweets or salty snacks. Trying to compensate, or what they called self-punishment -not eating at all or purging, perhaps reflect how much they really struggled with their eating behavior. Eating as a form of self-punishment is also reflected in previous findings [16].

### Experiencing lack of appetite and reduced capacity to plan and prepare meals

Some participants experienced losing appetite and hunger when stressed. The fact that stress affects both hunger and appetite is a known phenomenon. Yau and Potenza [19] writes that repeated and uncontrollable stress over time can dysregulate the hypothalamic–pituitary–adrenal (HPA) axis. In cases of chronical activation of the HPA-axis, the altered glucose metabolism can promote insulin resistance, and in this way influence appetite-related hormones and hypothalamic neuropeptides [24]. While noradrenaline and corticotropin-releasing factor may suppress appetite during stress, cortisol may stimulate appetite during recovery from stress [67]. In the present study, the participants described both increased and decreased appetite. In acute stress, the appetite is typically suppressed [25], whereas in chronic stress the desire for sweet and tasty high-fat foods is increased [24].

This fourth theme may differ from the above-stated three main themes; the participants described experiences of perceived stress in their daily life, and how this impacted on eating behaviors, without directly relate to previous trauma or traumatic stress. Despite this, the fourth theme was considered of importance and included. The participants experience about this theme was reported in all three focus groups, and it is conceivable that their experiences is indirectly connected with previous trauma, leading to lower current tolerance for and ability to cope with daily stress accompanied by changes in eating behaviors. For example, their experiences of exhaustion can be understood as posttraumatic stress symptoms, i.e., fatigue [68].

Further, if people with a history of trauma experience that perceived stress in everyday life impact eating behaviors by causing reduced capacity, including time and energy, to plan and prepare meals, leading to intake of ultra-processed food, sweet and salty snacks. This provides important knowledge for further understanding and research in the area. Despite that perceived stress in everyday life is far less dramatic than major traumatic life experiences, daily problems that accumulate, persist over time and affect special vulnerable areas of the individual [69], may have consequences for the individuals health [70]. However, an irregular diet and poor nutrition can also lead to loss of energy which might then generate a vicious circle of continuing to choose ultra-processed food, sweets and salty snacks. In addition, it seemed as if the participants lost the sense or meaning in life of eating healthy in periods of ‘unbearable’ stress.

## Limitations of the study

The present study using a qualitative approach, gives only an insight into a relatively small group of peoples’ experiences. Firstly, the small sample size and low heterogeneity of this group might not reflect all of the perspectives and nuanced information in this specific topic, and exploration should be conducted with care. Because of the relatively comprehensive research topic of our study, and no established theory used, final conclusions would require a larger sample size [33].

Secondly, since trauma history, referred to as the individuals’ subjective experience of trauma, was an inclusion criterion in the study, we did not systematically measure PTSD-symptoms. However, the aim of the present study was to explore how inpatients experience the impact of traumatic stress on eating behavior, regardless of specific types of traumatic events. Thirdly, none of the informants took advantage of the opportunity to read the transcriptions and have, therefore, not been given the opportunity to make any corrections or comments to what they have said, which thus may affect the validity of the results [71]. Including peer researchers in the interpretation of results strengthens qualitative methodology, which in this study resulted in a broader and deeper understanding of the data material through new insightful interpretations [35]. However, the peer researchers did not participate in the entire analysis process which may be considered a weakness in the sense that the authors may have overlooked important aspects and insights of the data material.

Fourthly, translation involves the risk of losing elements of meaning regarding the participants’ accounts. Additionally, using focus groups to obtain data about a sensitive subject like this might affect the depth of the stories of the participants. However, the aim of the study was to explore the field, and using focus group interviews enabled collection of much data in a relatively short time, in addition to the fact that the discussions facilitated important topics that the researchers had not foreseen.

## Conclusion

Consequences of traumatic experiences can be serious, affecting individuals in psychological and physiological, as well as behavioral ways. Evidently, the participants’ experiences in this present study show that after effects of traumatic life experience impacts eating behavior in several complex ways (Themes 1, 2, 3 and 4). These results contribute to the literature in providing an understanding of changes in eating behavior that might appear in people struggling with traumatic stress after traumatic experiences. This knowledge is important and useful for health professionals who offer help to people struggling with eating behavior after traumatic experiences since it emphasizes the need to better understand the complexity of eating behavior that over time may have a negative impact on health. However, these present observations call for further research, understanding how traumatic experiences can impact eating behavior, both in terms of being able to offer evidence-based help, but also to understand the connection between trauma and physical health conditions. Further research is needed with a focus on possible associations between trauma, eating behavior that can cause adverse health consequences, stress and disordered eating. Mapping trauma history and PTSD-symptoms and the occurrence or symptoms of EDs, whilst investigating the impact of traumatic stress on eating behaviors, might be a further line of enquiry.

## Data Availability

The datasets generated and analyzed during the study are not publicly available on the ground that public access to the data material was not included in the written consent form. Upon reasonable request, the data material is available in anonymized format from the corresponding author.

## Abbreviations

BN: Bulimia nervosa
BED: Binge-eating disorder
ED: Eating disorder
HPA: Hypothalamic–pituitary–adrenal
PTSD: Post traumatic stress disorder
STC: Systematic text condensation
